# Novel auxetic semiconductors with high carrier mobility: first principles prediction of Janus Ge_2_XY (X/Y = S, Se, Te) monolayers

**DOI:** 10.1039/d4na00852a

**Published:** 2025-02-21

**Authors:** Vo Q. Nha, Nguyen Q. San, Huynh T. T. Linh, Tuan V. Vu, Nguyen D. Hien

**Affiliations:** a School of Engineering and Technology, Hue University Hue Vietnam; b Laboratory for Computational Physics, Institute for Computational Science and Artificial Intelligence, Van Lang University Ho Chi Minh City Vietnam tuan.vu@vlu.edu.vn; c Faculty of Mechanical - Electrical and Computer Engineering, School of Technology, Van Lang University Ho Chi Minh City Vietnam; d Institute of Research and Development, Duy Tan University Da Nang Vietnam nguyendinhhien2@duytan.edu.vn; e School of Engineering & Technology, Duy Tan University Da Nang Vietnam

## Abstract

Recently, auxetic materials have attracted attention due to their unusual behavior and multifunctional applications. A negative Poisson's ratio has been found in some two-dimensional (2D) asymmetric layered materials. In this work, we predict a new class of 2D auxetic materials with the chemical formula Ge_2_XY (X/Y = S, Se, Te) using *ab initio* calculations. We construct the crystal structure and evaluate the stability of Janus Ge_2_XY monolayers under ambient conditions. Phonon dispersion spectra, cohesive energy calculations, and molecular dynamics simulations confirm the high structural stability of Ge_2_XY. At the ground state, Ge_2_XY monolayers are semiconductors with narrow band gaps ranging from 0.11 to 1.09 eV. We also calculate the mechanical properties, including elastic constants, Young's modulus, and Poisson's ratio. Importantly, the Ge_2_XY monolayers represent ideal auxetic materials with a large negative Poisson's ratio. All three Ge_2_XY systems possess Poisson's ratio values of around −0.2 along the *x*-axis. Moreover, Ge_2_XY monolayers are predicted to have high electron mobility up to 10.92 × 10^3^ cm^2^ V^−1^ s^−1^ (Ge_2_STe). The combination of ideal auxetic behavior and tunable transport properties makes the Janus Ge_2_XY structures promising materials for nanoelectronic and mechanical applications.

## Abstract 2

Recently, auxetic materials have attracted attention due to their unusual behavior and multifunctional applications.

## Graphical abstract

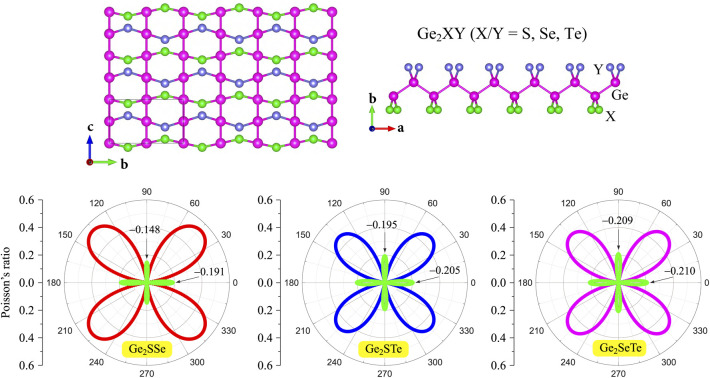

## 1 Introduction

One of the important parameters for determining a material's mechanical characteristics is Poisson's ratio. Auxetic materials, which possess a negative Poisson's ratio (NPR), have several unique and beneficial properties, such as high indentation resistance,^1^ improved toughness,^2^ and superior sound absorption.^3^ These enhanced features could lead to uses in protector devices,^4^ healthcare,^5^ and industry.^6^ Previous reports have shown that bulk, two-dimensional (2D), and even one-dimensional (1D) materials exhibit NPR effects. For example, 1D carbon nanostructures named diamond nanothreads show auxetic behavior.^7^ The NPR phenomena were also observed in poly[5]asterane,^8^ which is known to be a 1D nanostructure with mechanical and chemical stability. 3D structures, such as composites,^9^ microporous polymers,^10^ and cubic metals,^11^ also exhibit NPR effects. Like bulk and 1D materials, auxetic behavior is also present in many 2D nanostructures. Jiang and Park observed that the first 2D material to possess the NPR phenomenon was black phosphorus.^12^ However, the effect in black phosphorus is small, with a Poisson's ratio value of −0.027. Other 2D structures, such as δ-phosphorene,^13^ borophene,^14^ and penta-graphene,^15^ are auxetic materials. According to earlier results, the NPR behavior originates from the unusual geometrical structures of the materials, such as re-entrant or puckered crystal structures.

Very recently, the NPR phenomenon has also been observed in van der Waals heterostructures. Li and co-workers predicted that the graphene/hexagonal boron nitride (G/h-BN) superlattice in various stacking modes possesses NPR values of around −0.1.^16^ Interestingly, the authors showed that the auxetic behavior in the G/h-BN superlattice originates from the interaction of p_*z*_ orbitals between the interfacial layers. The strength of this interaction depends on the distance-dependent hopping integral, which is considered a parameter related to the electronic band structure. This study has contributed to enriching the family of auxetic materials. In addition, it has been shown that the NPR phenomenon is present in 2D Janus asymmetric materials. It is well known that the mirror symmetry structure is lost when we add a third element to the binary compound, forming an asymmetric configuration in the vertical direction. The symmetry breaking in Janus structures has given rise to many unusual and interesting properties compared to their symmetric counterparts.^17–19^ Therefore, 2D Janus structures have introduced a new class of materials with diverse applications in different fields.^20–24^ In recent studies, auxetic behavior has been found in Janus systems, further enriching the exotic properties of this class of materials. For example, Hiep *et al.* theoretically predicted that Janus Si_2_XY (X/Y = S, Se, Te) monolayers are stable structures possessing NPR effects along both in-plane directions.^25^ The Si_2_SSe monolayers exhibit the highest auxetic behavior, possessing a Poisson's ratio value of −0.131 along the *x*-axis. In addition, Si_2_OS and Si_2_OSe semiconductors have both been predicted to exhibit Poisson's ratio with large negative values.^26^ Amazingly, the NPR effect in Janus Si_2_OS monolayers is superior, up to −0.234 along the *x*-direction. The advantages of superior electronic properties and NPR effects make these Janus structures promising for applications in various fields. To date, few studies have explored the NPR effect in Janus structures, so it is worth searching for new auxetic materials belonging to the family of Janus materials.

Group 2D IV–VI compounds are known as representative materials in the family of 2D nanostructures because of their unique structure and physical properties.^27,28^ Yang *et al.* assumed that the *Pma*2-SiS semiconductor has a moderate bandgap.^29^ Remarkably, compared to 2D α-SiS and β-SiS, the smaller formation energy suggests that the 2D *Pma*2-SiS structure is more structurally stable. Furthermore, because of its appealing *ZT* values at medium-high temperatures, the 2D *Pma*2-SiSe monolayer has recently been suggested as a potential thermoelectric material.^30^ Previously, the first-principles technique revealed the mechanical and electrical characteristics of 2D SiS, SiSe, and Si_2_SSe.^31^ It has been predicted that these structures are good candidates for auxetic materials due to their high negative Poisson's ratio values. In particular, the SiS monolayer possesses a high NPR value of −0.19 in the *x*-axis.^31^

In addition to the auxetic behavior, 2D IV–VI structures possess high anisotropic carrier mobility and strain-tunable band gap energy, making them promising in optoelectronic and nanomechanical applications. The NPR phenomenon was also observed in GeS monolayers by using first-principles calculations.^32^ Also, piezoelectricity was enhanced in GeS compared to transition metal dichalcogenide and hexagonal BN monolayers. Intrigued by the diverse and outstanding physical properties of group IV–VI materials, herein, we investigate the structural, mechanical, electronic, and transport features of three 2D Janus Ge_2_XY (X/Y = S, Se, Te) monolayers employing *ab initio* calculations. The combination of remarkable transport properties and exotic auxetic behavior makes this family of materials a potential candidate for multifunctional applications.

## 2 Computational details and methodology

In the present manuscript, all simulations were carried out by density functional theory (DFT) *via* the Vienna *ab initio* simulation package (VASP)^33,34^ in conjunction with the projector augmented wave approach.^33^ We used the generalized-gradient approximation formulated by Perdew–Burke–Ernzerhof (PBE)^35^ to treat the electronic exchange–correlation energy. Further, the Heyd–Scuseria–Ernzerhof hybrid functional (HSE06) was used to achieve an accurate band structure.^36^ A *k*-points grid of (15 × 15 × 1) was adopted for integration over the Brillouin zone using the Monkhorst–Pack scheme.^37^ For the plane-wave expansion, a kinetic cutoff energy of 500 eV was applied. The convergence criteria of energy and forces on each atom are set to be 10^−6^ eV and 10^−3^ eV Å^−1^, respectively. A vacuum region of 20 Å was adopted in the *z* direction to reduce the interaction between neighboring slabs. *Ab initio* molecular dynamics (AIMD) simulations were employed to examine the thermal stability of structures.^38^ We recorded the phonon dispersion spectra by using the finite displacement technique *via* the PHONOPY code.^39^ Carrier mobility in the proposed materials is calculated based on deformation potential (DP) theory.^40^

## 3 Results and discussion

### 3.1 Crystal structure, structural stability, and mechanical features

**Fig. 1:**
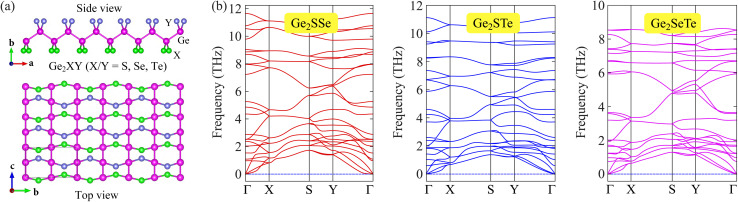
Crystal structures (a) and phonon dispersions (b) of Janus Ge_2_XY (X/Y = S, Se, and Te; X ≠ Y) monolayers.

We first examine the crystal structures of Janus Ge_2_XY monolayers and their structural stability. Fig. 1(a) reveals the optimized structure of the Janus Ge_2_XY monolayers from various views. Notably, Ge_2_XY has an orthorhombic structure. Indeed, there are eight atoms in the unit cell: two X, two Y, and four Ge atoms. We can obtain the asymmetric Ge_2_XY structure from its symmetric counterpart GeX by replacing one layer of X atoms with one layer of Y atoms. We calculate the structural parameters of Ge_2_XY crystals and summarize them in Table 1. Our results reveal that Janus Ge_2_SSe, Ge_2_STe, and Ge_2_SeTe monolayers have lattice constants a (b) of 6.98 Å (4.11 Å), 7.12 Å (4.14 Å), and 7.40 Å (4.17 Å), respectively. The increasing trend of the lattice constant is consistent with the increasing atomic radius of the chalcogen element in the periodic table. At the same time, the bond length between two neighboring Ge atoms is almost unchanged in all three predicted structures. Meanwhile, the Ge–X and Ge–Y bond lengths depend on the atomic radius of the chalcogen element, similar to the changing trend of the lattice constant.

**Table 1:** Optimized lattice constants *a* and *b*, bond length *d*, cohesive energy *E*_c_, and elastic constant *C*_*ij*_ of Ge_2_XY monolayers

	*a* (Å)	*b* (Å)	*d*_Ge–X_ (Å)	*d*_Ge–Y_ (Å)	*d*_Ge–Ge_ (Å)	*E*_c_ (eV per atom)	*C*_11_ (N m^−1^)	*C*_12_ (N m^−1^)	*C*_22_ (N m^−1^)	*C*_66_ (N m^−1^)
Ge_2_SSe	6.98	4.11	2.26	2.38	2.48	4.47	61.03	−9.01	47.22	6.57
Ge_2_STe	7.12	4.14	2.28	2.60	2.50	4.28	45.39	−8.84	43.12	6.40
Ge_2_SeTe	7.40	4.17	2.42	2.60	2.50	4.14	42.85	−8.96	42.73	5.83

Next, we examine the stability of the proposed structures. We first calculate the cohesive energies of the proposed structures to evaluate the strength of their chemical bond lengths. The cohesive per atom *E*_c_ of Janus Ge_2_XY can be calculated as^41^1
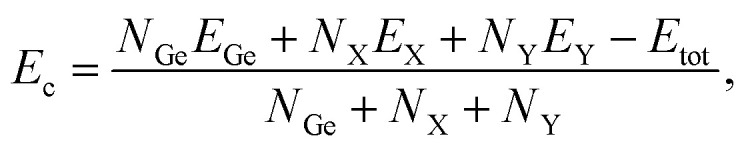
where *N*_Ge_, *N*_X_, and *N*_Y_ are the number of atoms Ge, X, and Y in the primitive cell, respectively; *E*_Ge_, *E*_X_, and *E*_Y_ refer to the energies of the single atoms Ge, X, and Y, respectively; *E*_tot_ stands for the total energy of the proposed structures. The calculated cohesive energies of Janus Ge_2_SSe, Ge_2_STe, and Ge_2_SeTe monolayers are found to be 4.47, 4.28, and 4.14 eV per atom, as listed in Table 1. With large cohesive energy, the proposed materials are energetically stable with strong internal bonds. It is shown that the cohesive energy of Janus Ge_2_XY decreases with increasing lattice constant. Obviously, the greater the distance between atoms, the weaker the bond between atoms. The cohesive energies of Janus Ge_2_XY monolayers are comparable with those of similar structures, including Janus γ-Ge_2_SSe (3.55 eV per atom),^41^ γ-Sn_2_SSe (4.18 eV per atom),^28^ Si_2_OS (4.89 eV per atom)^42^ or Si_2_SSe (5.10 eV per atom).^25^

To confirm the dynamical stability of the Janus Ge_2_XY structures, we recorded their phonon dispersion spectra along the high symmetry points in the Brillouin zone (BZ), as shown in Fig. 1(b). We can observe that the vibrational branches are all positive in the BZ for our three considered monolayers. It suggests that the Janus Ge_2_XY structures possess high dynamical stability. Fig. 1(b) reveals that the phonon spectrum contains 24 vibrational modes, including three acoustic and 21 optical branches. It is attributed to the presence of eight atoms in the unit cell.

**Fig. 2:**
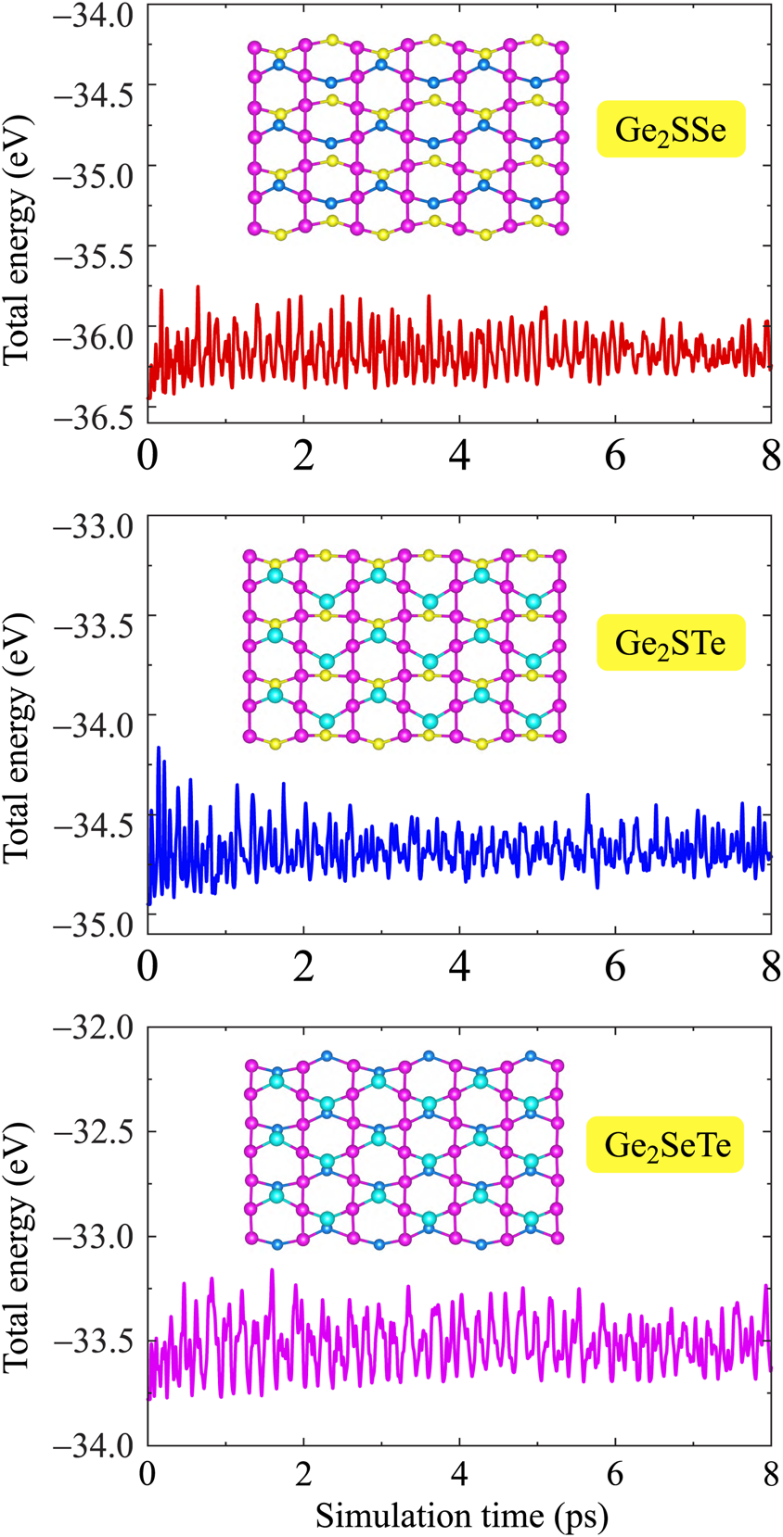
Time dependence of the total energies of Ge_2_XY from AIMD simulations at room temperature. Insets show the crystal structures of Ge_2_XY at 8 ps.

Along with the dynamical stability, we further consider the thermal stability of the Janus Ge_2_XY monolayers by performing AIMD simulations at room temperature within 8 ps. Fig. 2 plots the total energy fluctuations with the simulation time of Janus Ge_2_XY at room temperature. We note that the energy fluctuations occur only over a small range. The crystal structures of the calculated monolayers remain stable within 8 ps of the AIMD test. We observe no structural phase transitions or bond breaking in these monolayers after test. These results indicate the high thermal stability of the three Ge_2_XY structures.

Next, we explore the mechanical features of the Ge_2_XY structures, including the elastic constant *C*_*ij*_, Young's modulus *Y*_2D_, and Poisson's ratio 
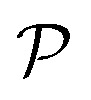
. For Ge_2_XY structures, there are four coefficients, including *C*_11_, *C*_12_, *C*_22_, and *C*_66_, as listed in Table 1. Our calculated results demonstrate that *C*_12_ has a negative value as revealed in Table 1. The presence of a negative value for *C*_12_ may lead to the auxetic behavior. Importantly, the obtained results satisfy the conditions of *C*_66_ > 0 and *C*_11_*C*_22_ − *C*_12_^2^ > 0, confirming the mechanical stability of Janus Ge_2_XY structures according to the Born–Huang criteria.^43,44^ Our calculated results demonstrated that Janus Ge_2_XY monolayers are dynamically, thermally, energetically, and mechanically stable. This supports the hypothesis that the proposed monolayers can be fabricated by conventional experimental methods.

The angle-dependent Young's modulus *Y*_2D_(*θ*) and Poisson's ratio 
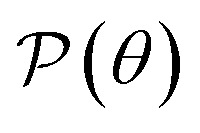
 are determined as follows:^45,46^2
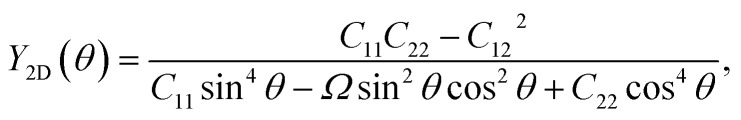
3
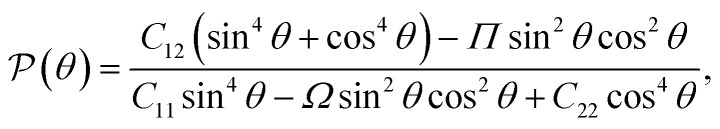
where *Ω* = 2*C*_12_ + (*C*_12_^2^ − *C*_11_*C*_22_)/*C*_66_ and *Π* = *C*_11_ + *C*_22_ + (*C*_12_^2^ − *C*_11_*C*_22_)/*C*_66_. The angle between the *x*-axis and examined direction is denoted by *θ*.

**Fig. 3:**
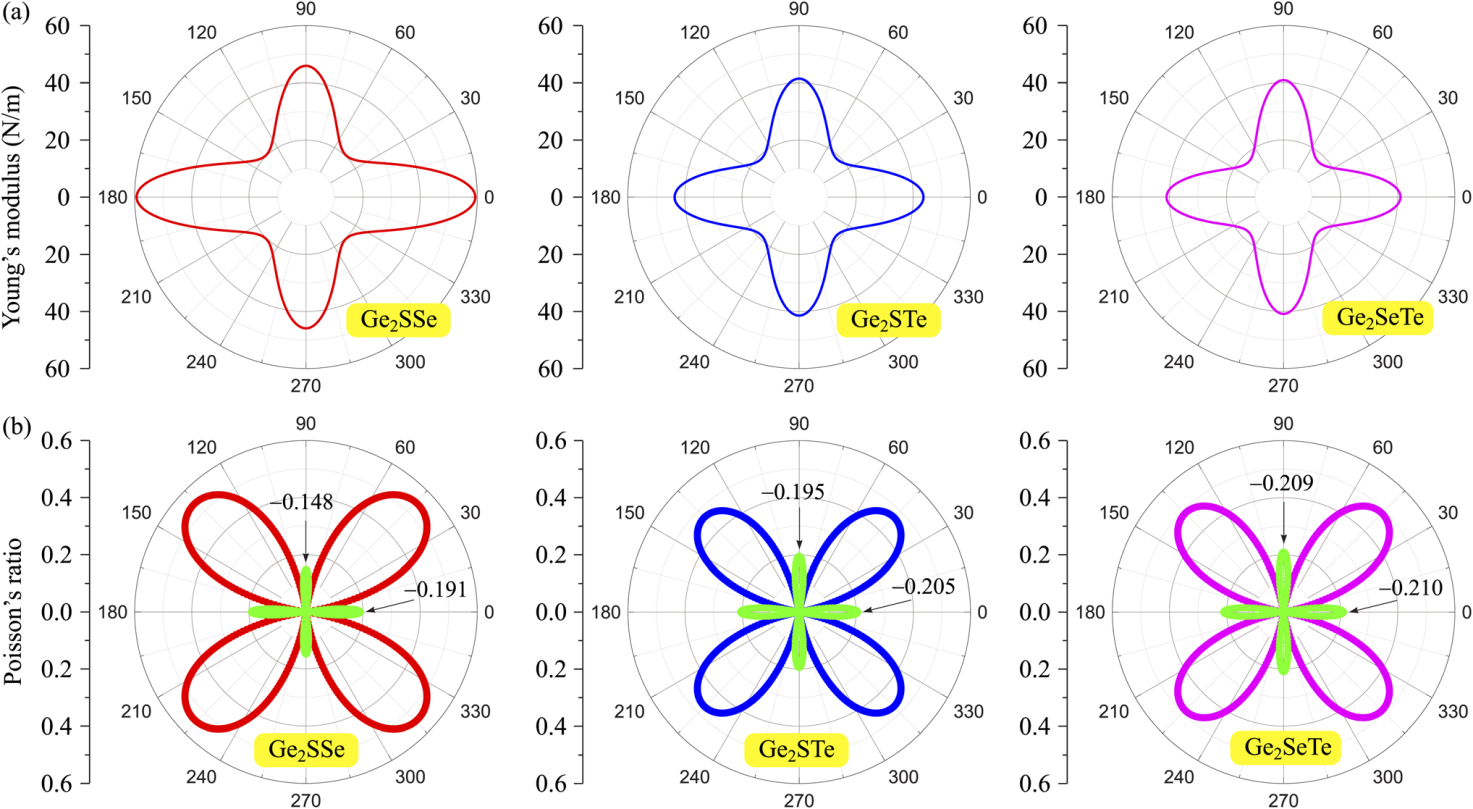
Young's moduli (a) and Poisson's ratios (b) of Janus Ge_2_XY monolayers. Negative values of Poisson's ratio in (b) are indicated in green.

Fig. 3(a) demonstrates the angle-dependent *Y*_2D_(*θ*) of the 2D Janus Ge_2_XY systems. As can be seen, Young's modulus exhibits a highly anisotropic character, consistent with the in-plane anisotropy of the crystal structure in the predicted monolayers. For all Ge_2_XY monolayers, *Y*_2D_ reaches its maximum value at *θ* = 0° and then decreases to a minimum value at *θ* = 45°. For example, the Ge_2_SeTe monolayer has a maximum *Y*_2D_ of 40.97 N m^−1^ at *θ* = 0° and a minimum *Y*_2D_ of 17.34 N m^−1^ at *θ* = 45°. This leads us to conclude that, for Janus Ge_2_XY materials, they will be stiffest along the zigzag direction (*θ* = 0°) and that they will be softest corresponding to *θ* = 45° and *θ* = 135°. In addition, the obtained value of Young's modulus decreases in the order from Ge_2_SSe to Ge_2_STe to Ge_2_SeTe. These calculated results can be explained by the fact that the shorter the interatomic bond length, the stronger the bond, and the higher the in-plane stiffness. Compared with other 2D structures, such as γ-GeS (73.25 N m^−1^),^41^ WS_2_ (137 N m^−1^)^47^ or graphene (336 N m^−1^)^48^ monolayer, the Young's modulus value of the 2D Ge_2_XY monolayer is much smaller. It implies that our predicted systems withstand applied strain better than other 2D systems.

Fig. 3(b) depicts the angle dependence of Poisson's ratio for the Janus Ge_2_XY systems. Like Young's modulus, Poisson's ratios of Ge_2_XY structures exhibit a highly anisotropic feature. As shown in Fig. 3(b), the angle-dependent 
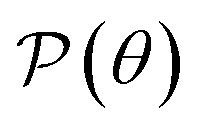
 plots of all three materials are similar. The maximum values of 
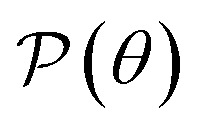
 for Ge_2_XY monolayers correspond to *θ* = 45° and *θ* = 135°. The maximum values of 
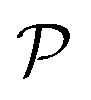
 for Ge_2_SSe, Ge_2_STe, and Ge_2_SeTe are calculated to be 0.54, 0.47, and 0.49, respectively. Importantly, we found the NPR along the two in-plane axes *x* and *y* in the polar diagrams of 
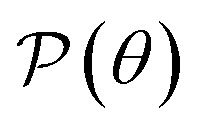
 of Ge_2_XY materials. It is indicated that Janus Ge_2_SeTe possesses the largest NPR of −2.10 along the *x* direction, as revealed in Fig. 3. The value of NPR along the *y* direction for Ge_2_SeTe is found to be −2.09. The NPR values along the *x*(*y*) direction are found to be −0.191(−0.148) and −0.205(−0.195) for Ge_2_SSe and Ge_2_STe, respectively. The negative Poisson's ratio behavior in Janus Ge_2_XY is closely related to its hinge-like structure. This hinge-like structure is a necessary condition for the negative Poisson's ratio. This has also been shown in phosphorene with a high negative Poisson's ratio.^49^ Possessing a high negative Poisson's ratio value, Janus Ge_2_XY monolayers exhibit a much stronger auxetic effect than other 2D auxetic structures, such as SiS (−0.19),^31^ borophene (−0.053),^49^ phosphorene (−0.027),^50^ or GeS monolayer (−0.137).^32^ The superior mechanical properties of Janus Ge_2_XY monolayers may lead to many nanomechanical applications.

### 3.2 Electronic characteristics

**Fig. 4:**
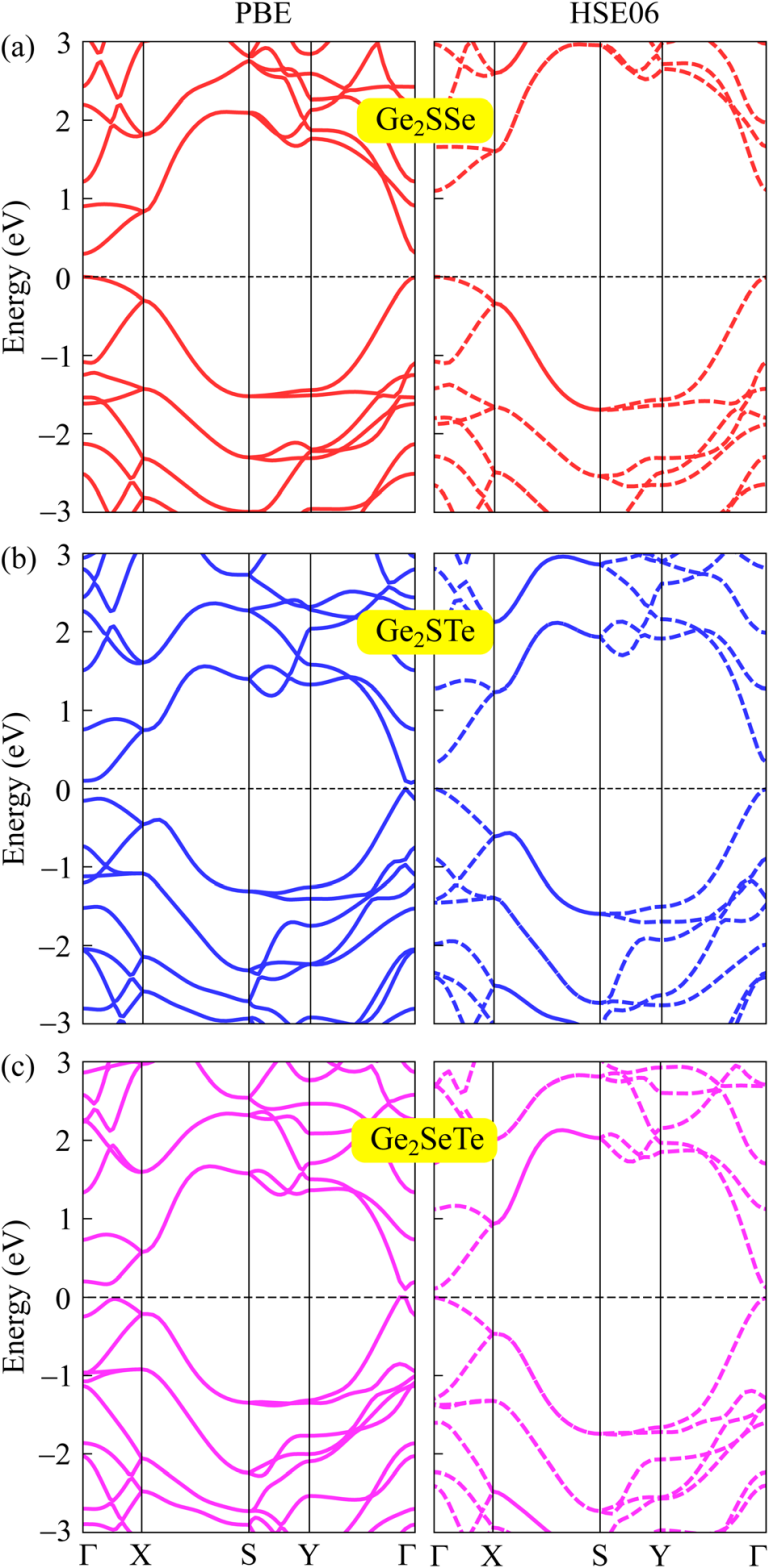
The computed band structures of Ge_2_SSe (a), Ge_2_STe (b), and Ge_2_SeTe (c) materials at the PBE (left) and HSE06 (right) theoretical levels.

In the following, we explore the electronic features of 2D Janus Ge_2_XY monolayers to suggest them for suitable applications. We use different functionals, namely PBE and HSE06, to calculate the electronic energy band structure of Ge_2_XY as illustrated in Fig. 4. Both methods reveal that the 2D Ge_2_XY systems are all semiconductors with small bandgaps. Compared to the PBE method, the HSE06 method provides higher bandgap values. Among all the proposed monolayers, the Ge_2_SSe monolayer possesses the widest bandgap energy of 1.09 eV at the HSE06 level. The other two monolayers have narrower HSE06 bandgaps, which are 0.32 eV and 0.11 eV for Ge_2_STe and Ge_2_SeTe, respectively. The obtained bandgap energies of Ge_2_XY are smaller than those of the Janus Si_2_SSe monolayer (1.51 eV) at the HSE06 level.^31^Table 2 summaries the obtained PBE and HSE06 bandgaps of Janus Ge_2_XY structures.

**Table 2:** Obtained PBE/HSE06 band gaps *E*_g_, difference between the vacuum levels Δ*Φ*, and work function on the X(Y) side *Φ*_X_(*Φ*_Y_) of Ge_2_XY materials

	*E* ^PBE^_g_ (eV)	*E* ^HSE06^_g_ (eV)	*Φ*_X_ (eV)	*Φ*_Y_ (eV)	Δ*Φ* (eV)
Ge_2_SSe	0.29	1.09	5.24	4.84	0.40
Ge_2_STe	0.07	0.32	5.46	4.57	0.89
Ge_2_SeTe	0.10	0.11	4.88	4.39	0.49

**Fig. 5:**
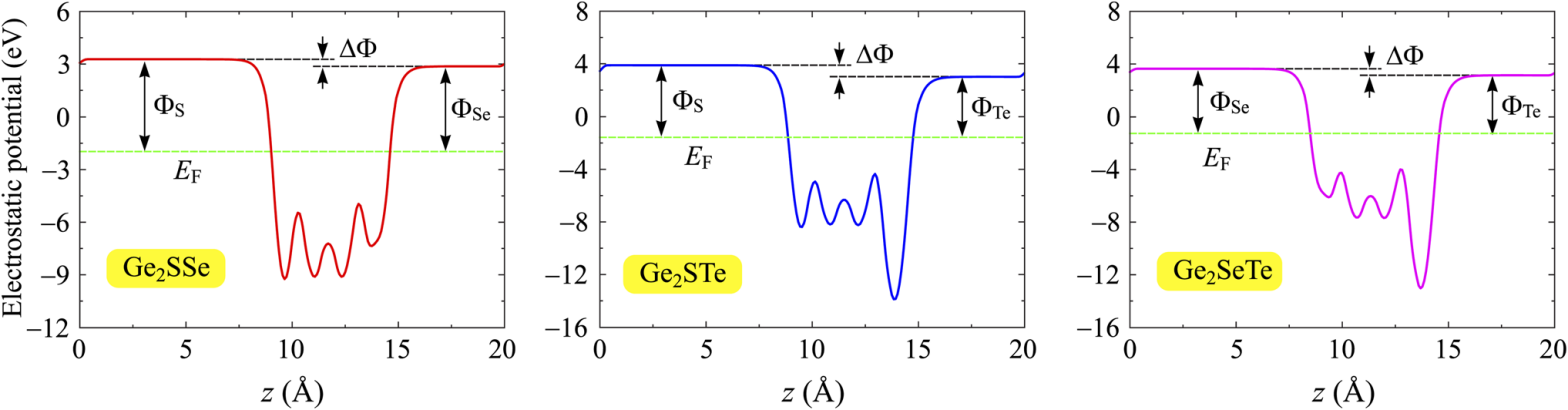
Electrostatic potentials of Ge_2_SSe, Ge_2_STe, and Ge_2_SeTe monolayers. The dashed horizontal lines indicate the Fermi level *E*_F_. The potential difference between the sides is labeled with ΔΦ.

In addition to the band structures, we calculate the electrostatic potentials and the work functions of Janus Ge_2_XY monolayers. The value of the work function reflects the energy required for electrons to escape from the crystal surface. The work function *Φ* is related to the Fermi level *E*_F_ and the vacuum level *E*_vac_ through the expression *Φ* = *E*_vac_ − *E*_F_. For the studied compounds, the two faces are made up of various chalcogen elements, whose electronegativities are different, giving rise to an out-of-plane dipole moment. The magnitude of this dipole moment depends on the electronegativity difference between the two faces of the material. Therefore, it is necessary to add the dipole correction when evaluating the electrostatic potentials in asymmetric structures.^51^Fig. 5 shows the electrostatic potentials of Janus Ge_2_XY monolayers with dipole corrections. The computed results reveal that the vacuum levels on the two faces of the structure are different, and the shape of the electrostatic potential is asymmetric. That is attributed to the lack of vertical mirror symmetry in the Janus structure. The values of the work function *Φ* and the vacuum level differences Δ*Φ* at the Ge_2_XY surfaces are listed in Table 2. For Ge_2_XY monolayers, the work functions at the X surface vary from 4.88 to 5.46 eV, while those at the Y surface range from 4.39 to 4.84 eV. Our results reflect that the energy required for electrons to escape from the Y surface is lower than that of the X surface. In addition, Table 2 also reveals that the Ge_2_STe monolayer has the highest vacuum level difference Δ*Φ* compared to the other monolayers. This result is consistent with the fact that the higher the electronegativity difference between the elements, the higher the vacuum level difference between the surfaces.

### 3.3 Transport features and carrier mobility

In the last part, we explore the fundamentals of the proposed Janus Ge_2_XY monolayers. Transport parameters, particularly carrier mobility, play a critical role in the performance and efficiency of electronic devices. It is necessary to investigate electron mobility because it strongly affects the efficiency, speed, and behavior of electronic devices. Here, we use deformation potential (DP) theory to explore the carrier mobility of the proposed structures.^40^

The carrier mobility in the framework of DP for 2D nanostructures is given by^52^4
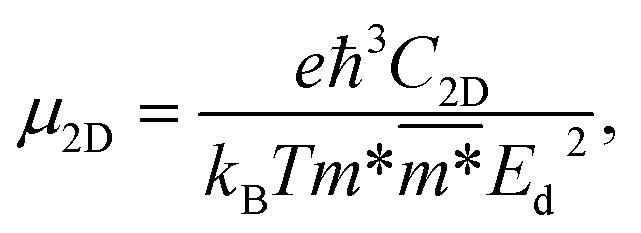
where *e* denotes the elementary charge, ℏ is the reduced Planck constant, *C*_2D_ refers to the elastic modulus, *k*_B_ is the Boltzmann constant, *T* = 300 K is the selected temperature, the effective mass is denoted by m*, the average effective mass is denoted by 
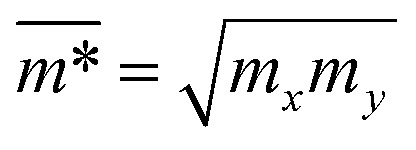
, and *E*_d_ is the DP constant.

The transport parameters can be calculated based on the DFT method *via* the following equations:5
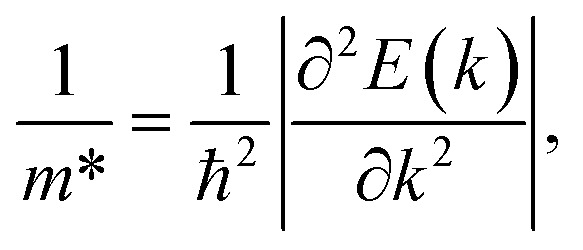
6
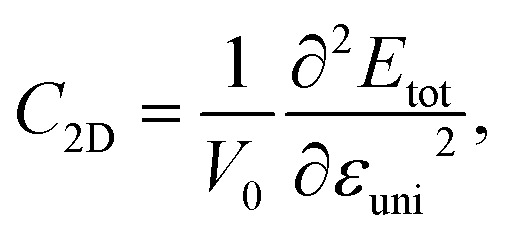
7
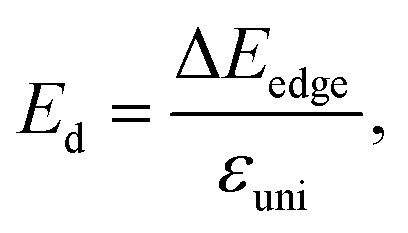
where *E*(*k*) stands for the wavevector (*k*) dependence of the band-edge energy, the area of the optimized cell is denoted by *V*_0_, the total energy of the 2D sheet is denoted by *E*_tot_, *ε*_uni_ indicates the applied uniaxial strain along the *x*/*y*-axis, and Δ*E*_edge_ is the band-edge (VBM/CBM) shifting relative to the vacuum level.

**Fig. 6:**
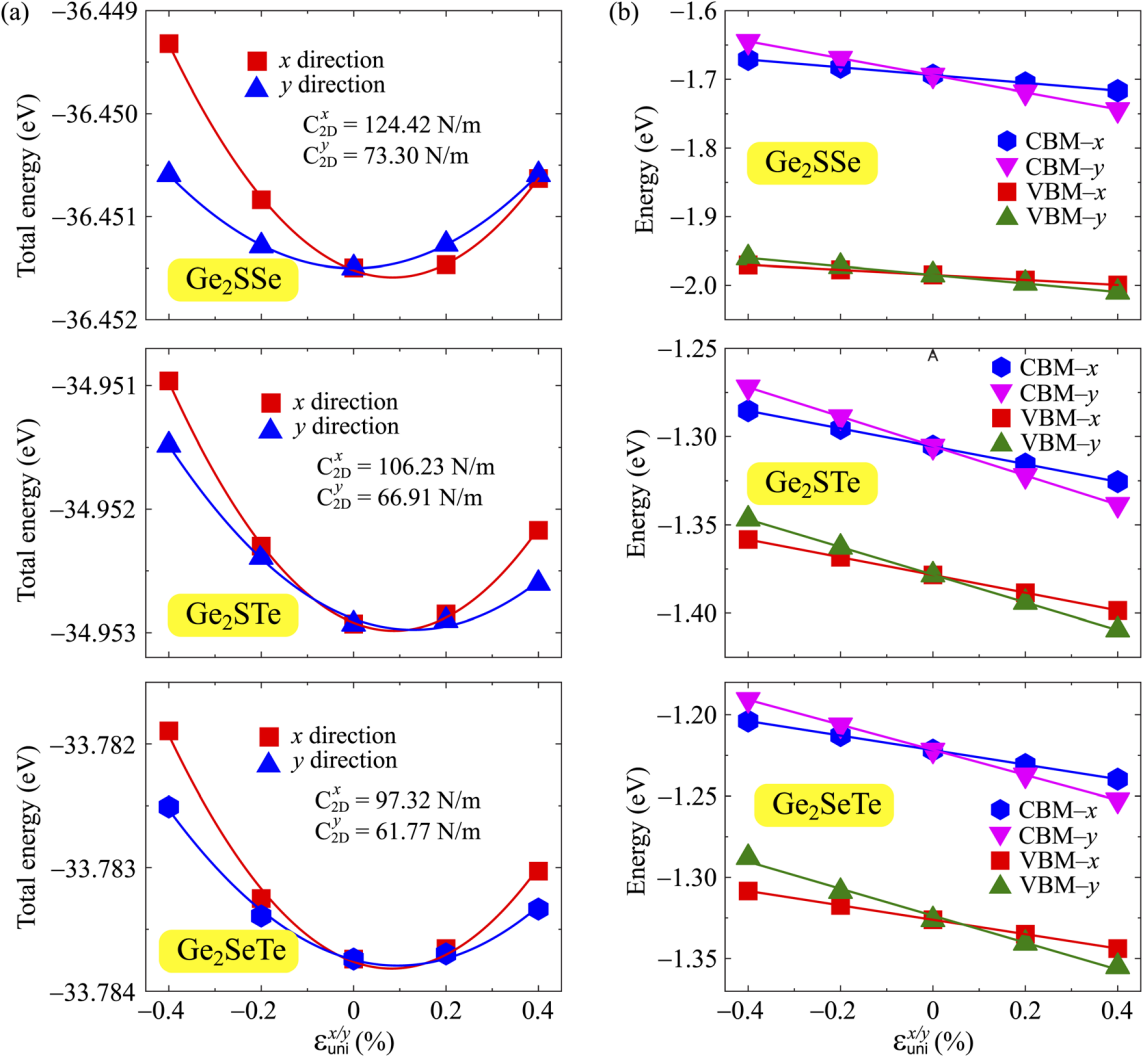
The uniaxial strain dependence of (a) the total energy shifts and (b) band-edge positions of Ge_2_XY structures.

The effective mass of charge carriers (holes and electrons) plays a fundamental role in determining carrier mobility in semiconductors. The effective mass is a concept that arises from the band structure and strongly affected by the curvature of the sub-band at the band-edges. From eqn (5), we can see that a flat curvature (large radius) indicates a large effective mass, while a steep curvature leads to a smaller effective mass. The calculated results for m* along the *x* and *y* axes are listed in Table 3. It can be seen that Janus Ge_2_XY monolayers exhibit very low electron-effective mass. Further, the effective mass of carriers exhibits highly directional anisotropy along the two transport directions *x* and *y*. For example, the calculated values of *m*_*x*_ and *m*_*y*_ for Janus Ge_2_SSe are calculated to be 0.67*m*_0_ and 0.13*m*_0_ (*m*_0_ refers to the mass of the free electron), respectively. We can obtain the *C*_2D_ and *E*_d_ by fitting the uniaxial strain dependence of the total energy and band-edge positions as shown in Fig. 6. In these procedures, a small uniaxial strain *ε*^*x*/*y*^_uni_ ranging from −0.4 to 0.4% is applied along the *x*/*y* axis. The computed values of *C*_2D_, *E*_d_, and corresponding *μ*^*x*/*y*^_2D_ are listed in Table 3. We can see that the transport parameters of Ge_2_XY monolayers exhibit highly directional anisotropic characteristics. This is consistent with the anisotropy in the crystal structures of Ge_2_XY monolayers. It is demonstrated that Ge_2_XY monolayers have high electron mobility, as shown in Table 3. The electron mobility along the *x* (*μ*^*x*^_2D_) and *y* (*μ*^*y*^_2D_) directions for Ge_2_SSe is 417.79 and 266.44 cm^2^ V^−1^ s^−1^, respectively. The Janus Ge_2_SeTe possesses a higher electron mobility with *μ*^*x*^_2D_ = 5.57 × 10^3^ cm^2^ V^−1^ s^−1^ and *μ*^*y*^_2D_ = 501.38 cm^2^ V^−1^ s^−1^. Particularly, ultra-high electron mobility up to 10.92 × 10^3^ cm^2^ V^−1^ s^−1^ is found along the *x* axis in the Janus Ge_2_STe monolayer as presented in Table 3. With high electron mobility, Janus Ge_2_XY monolayers are suitable for applications in flexible nanoelectronics.

**Table 3:** Effective mass m* (*m*_0_), DP constant *E*_d_ (eV), elastic modulus *C*_2D_ (N m^−1^), and mobility of carriers *μ*_2D_ (cm^2^ V^−1^ s^−1^) of Janus Ge_2_XY monolayers along the *x* and *y* directions. *m*_0_ is the mass of the free electron

		*m*_*x*_	*m*_*y*_	*C* ^*x*^_2D_	*C* ^*y*^_2D_	*E* ^*x*^_d_	*E* ^*y*^_d_	*μ* ^*x*^_2D_	*μ* ^*y*^_2D_
Electron	Ge_2_SSe	0.67	0.13	124.42	73.30	−5.66	−12.35	417.79	266.44
	Ge_2_STe	0.10	0.07	106.23	66.91	−5.01	−8.31	10.92 × 10^3^	3.68 × 10^3^
	Ge_2_SeTe	0.11	0.26	97.32	61.77	−4.47	−7.72	5.57 × 10^3^	501.38
Hole	Ge_2_SSe	2.70	0.31	124.42	73.30	−3.62	−6.18	81.75	143.94
	Ge_2_STe	0.17	0.18	106.23	66.91	−5.02	−7.84	3.02 × 10^3^	735.37
	Ge_2_SeTe	0.09	0.07	97.32	61.77	−4.43	−8.30	14.50 × 10^3^	3.97 × 10^3^

## 4 Conclusion

In conclusion, a new class of 2D auxetic materials Ge_2_XY (X/Y = S, Se, Te) has been predicted by employing *ab initio* calculations. Janus Ge_2_XY monolayers have been predicted to be stable crystalline semiconductors. They are materials with great mechanical flexibility due to their small Young's coefficient. Due to the anisotropic crystalline structure, the mechanical properties of Ge_2_XY are highly anisotropic. In particular, they possess auxetic behaviors with large negative Poisson's ratios, up to −0.210 (Ge_2_SeTe monolayer). We found that the auxetic effect along the *x*-axis is the strongest. The transport characteristics of Ge_2_XY monolayers also exhibit strong anisotropic characteristics. The carrier mobility along the *x*-axis is computed to be larger than that along the *y*-axis. With their large carrier mobility and outstanding mechanical properties, 2D Janus Ge_2_XY monolayers have great prospects for applications in flexible nanoelectromechanical devices. Our findings broaden the range of auxetic materials and provide insight into the physical characteristics of 2D Ge_2_XY structures.

## Data availability

All data that support the findings of this study are included within the article.

## Conflicts of interest

There are no conflicts of interest to declare.
